# Pressure gradient measurement using phase contrast (PC)-MRI in stenotic phantom models: Towards noninvasive quantification of fractional flow reserve in the coronary arteries

**DOI:** 10.1186/1532-429X-18-S1-W15

**Published:** 2016-01-27

**Authors:** Zixin Deng, Sang Eun Lee, Zhaoyang Fan, Christopher T Nguyen, Qi Yang, Xiaoming Bi, Byoung Wook Choi, Daniel S Berman, Hyuk-Jae Chang, Debiao Li

**Affiliations:** 1 Cedars Sinai Medical Center, Los Angeles, CA USA; 2 Bioengineering, University of California, Los Angeles, Los Angeles, CA USA; 3 Severance Cardiovascular Hospital, Yonsei College of Medicine, Seoul, Korea (the Republic of); 4 MR R&D, Siemens Healthcare, Los Angeles, CA USA; 5 Radiology, Research Institute of Radiological Science, Yonsei University College of Medicine, Seoul, Korea (the Republic of)

## Background

Fractional flow reserve (FFR) is an invasive diagnostic tool to evaluate the functional significance of an intermediate coronary stenosis by quantifying the pressure gradient (ΔP) across that stenosis [1]. Noninvasive ΔP measurement (ΔP_MR_) using phase-contrast (PC)-MRI in conjunction with Navier-Stokes (NS) equations has been attempted in large to medium size vessels [2–4], and recently been applied to smaller coronary arteries [5]. However, the measurement accuracy awaits systematic validation given that the small, tortuous, and moving caliber in the coronary artery, particularly when a stenosis exists, may elicit errors in flow velocity quantification by PC-MRI. This study aimed to investigate the feasibility of deriving ΔP_MR_ in small caliber stenotic phantom models at various diameters and its correlation with measured ΔP values via a pressure transducer (ΔP_PT_).

## Methods

**Figure 1:**
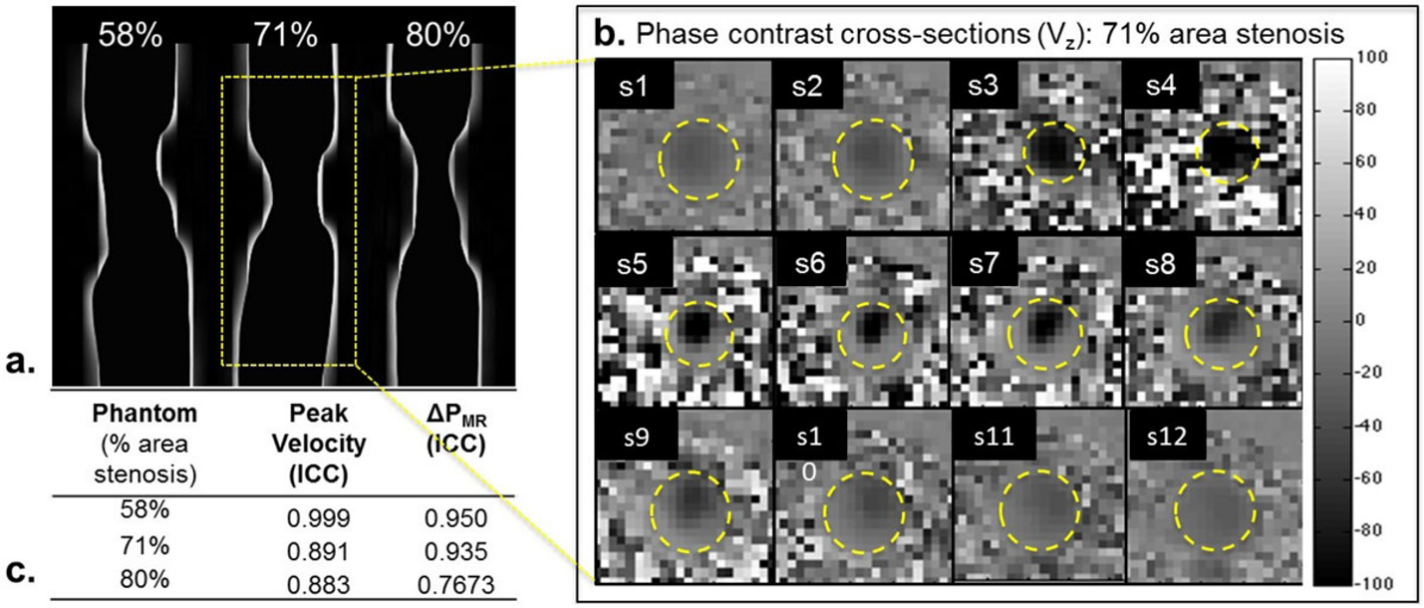
**a Stenotic phantom model examples (% area stenosis at the maximum narrowing)**. **b** 2D PC-MRI images in the through-plane direction (velocity maps, cm/s) for 71% area stenosis phantom model. **c** Intra-class correlation coefficients (ICC) of the peak velocities and ΔP_MR_s for the example phantom models.

11 small caliber phantom models ranging from 0%-85% area stenosis, with a reference diameter of 4.8 mm, were individually connected to a flow pump (gadolinium-doped water, constant volume velocity = 250 mL/min) while 2D cross-sectional PC-MRI images were acquired. Contiguous slices (10-20) were consecutively collected across each narrowing (fig. 1a/b). Imaging parameters were: FA = 15°; TE/TR = ~4.0/70 ms; in-plane spatial resolution = ~0.55 × 0.55 mm^2^; slice thickness = 3.2 mm; V_enc_ = z (40-260 cm/s) and x, y (40-80 cm/s), depending on the degree of narrowing. Eddy-current correction was done offline followed by NS calculations [6]. Repeat scans were performed in 7/11 phantom models and reproducibility was assessed by calculating the intra-class correlation coefficient (ICC) and Bland-Altman plots. Immediately following the PC-MRI scans, pressure was measured using an arterial catheter connected to a pressure transducer at ~1.5 cm before and ~3 cm after the maximum narrowing of the phantom models.

## Results

**Figure 2:**
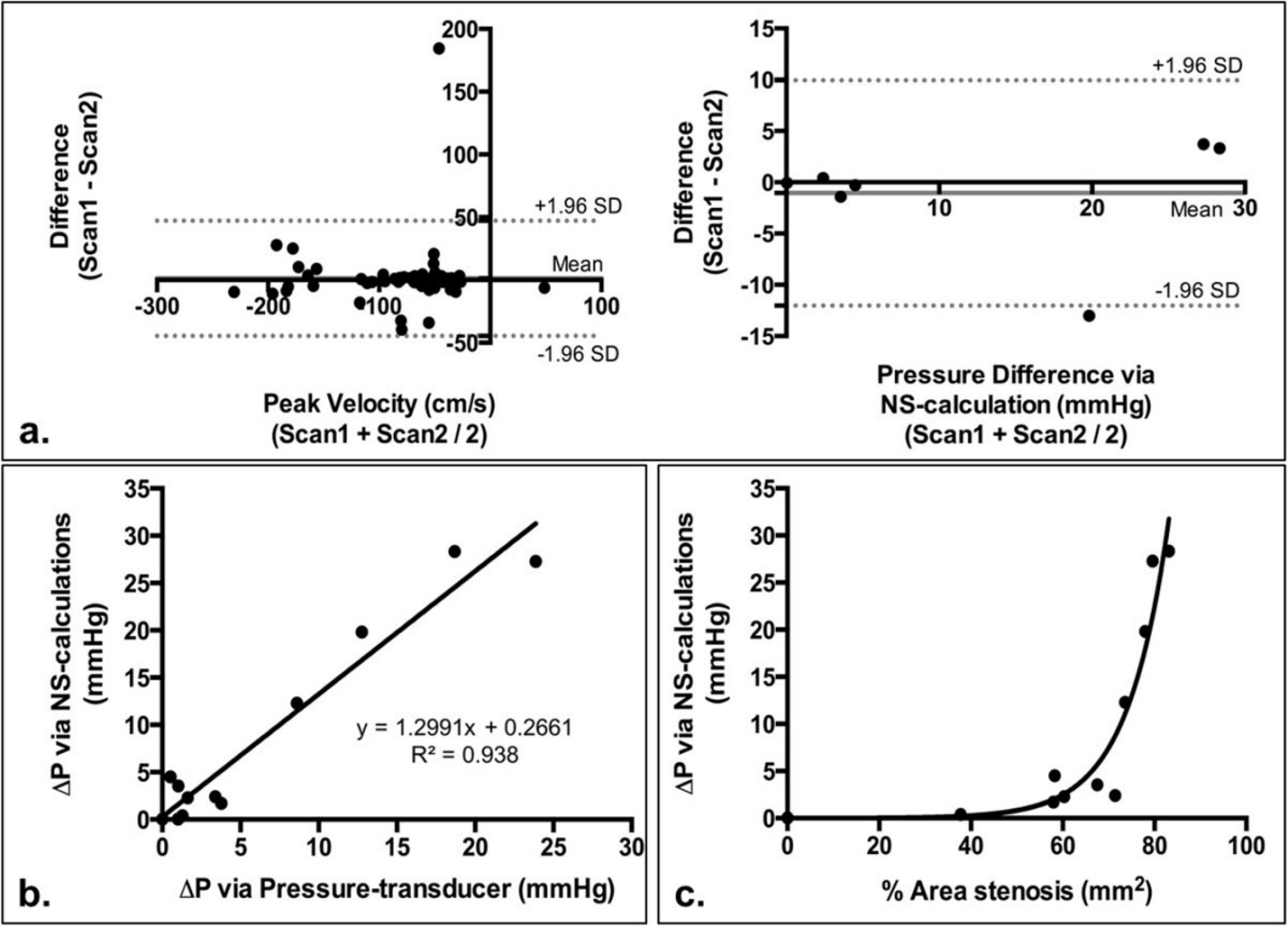
**a Bland-Altman plots of peak velocities at all cross-sectional slice from repeat PC-MRI scans and the derived ΔP of the phantom models**. Mean (bias) and 95% confidence internal limits are displayed. **b** Pressure measurement comparison between ΔP calculated via NS-equations and ΔP measured using pressure transducer. Excellent correlation (R^2^ = 0.938) was observed between the two techniques. **c** % area stenosis versus ΔP_MR_ measurement. An exponential increase in ΔP_MR_ was observed as % area stenosis increases.

Bland-Altman plots of peak velocities and ΔP_MR_ are shown in fig. 2a. For velocity measurements, excellent correlation was seen in the through-plane peak velocities (Vz, ICC = 0.90) and lower in Vx (ICC = 0.57) and Vy (ICC = 0.58). For ΔP_MR_s, overall ICC = 0.87; When observed individually, higher correlation was seen at smaller stenosis degrees and weaker as stenosis increased (fig. 1b). This could be due to the increased velocity in larger stenosis, causing minor turbulence distal of the narrowing, thus, inconsistent velocity and ΔP_MR_ between the two scans. Furthermore, ΔP_MR_ and ΔP_PT_ were highly correlated (fig. 2b). We also observed that as % area stenosis increased, ΔP_MR_ also increased (fig. 2c).

## Conclusions

Preliminary results suggest that quantification of ΔP_MR_ in a small caliber is feasible. Further technical improvements in higher in-plane and through-plane spatial resolutions and reduction of noise need to be employed, which could potentially help improve the accuracy of the ΔP_MR_ calculations.
